# Supplementary material for: Climate velocities and species tracking in global mountain regions
Source: Nature. 2024 Mar 27;629(8010):114–20. doi: 10.1038/s41586-024-07264-9 (PMC11062926; doi:10.1038/s41586-024-07264-9)
Supplement: Supplementary file 1 — A file contains Supplementary Methods, Supplementary Results and Supplementary Figures. [file 41586_2024_7264_MOESM1_ESM.pdf]

nature portfolio

https://doi.org/10.1038/s41586-024-07264-9

Supplementary information

Climate velocities and species tracking in global mountain regions

In the format provided by the authors and unedited

Supplementary Information of

Climate Velocities and Species Tracking in Global Mountain Regions

Wei-Ping Chan[1,2,3,4], Jonathan Lenoir[5], Guan-Shuo Mai[1], Hung-Chi Kuo[6], I-Ching Chen[7,8]*, and Sheng-Feng Shen[1]*

[1]Biodiversity Research Center, Academia Sinica, Taipei, 11529, Taiwan

[2]Department of Organismic and Evolutionary Biology, Harvard University, Cambridge, MA 02138, USA

[3]Bachelor Program in Data Science and Management, Taipei Medical University, Taipei, 106, Taiwan

[4]Rowland Institute at Harvard University, Cambridge, MA 02142, USA

[5]UMR CNRS 7058, Ecologie et Dynamique des Systèmes Anthropisés (EDYSAN), Université de Picardie Jules Verne, Amiens, France

[6]Department of Atmospheric Sciences, National Taiwan University, Taipei, 10617, Taiwan

[7]Department of Life Sciences, National Cheng Kung University, Tainan, 70101, Taiwan

[8]Department of Biology, Stanford University, Stanford, CA 94305, USA

*Corresponding author. Email: shensf@sinica.edu.tw, chenic@ncku.edu.tw

Supplementary Methods

Barometric formula

$$p = p_b \times exp\left[\frac{-g \times M \times h}{R \times T}\right]$$

where p_b denotes the static pressure (101,325.00 pascals), M denotes the molar mass of Earth's air (0.0289644 kg/mol), R denotes the universal gas constant for air (8.31432 N m mol^{-1} K^{-1}), h denotes the elevation above sea level (meters), and T denotes the reference temperature (288.15 K)

Biological datasets and corresponding climate velocities

We used a carefully curated range shift dataset[4], replacing latitude with absolute latitude in the analysis. For each biological record, climate velocity, geographic, topographic, and climatic variables were extracted according to coordinates. To maintain focus and stay within the range of studies of biological interest, second-level climate variables generated from published datasets are often used without further validation[61,62]. For example, seasonal precipitation calculated from published monthly data was used for biological studies without further checks[62]. Therefore, the idea of validating MALRT/SLRT in this study should be considered to understand their relationship and to explore the relative importance of the two variables on the range shifts of species, which can be explored by the random forest machine learning method. Topographic data— such as aspect, evenness, homogeneity, profile curvature, roughness, slope, tangential curvature, and terrain ruggedness— were derived from EarthEnv[54] at a resolution of 0.5 degrees (~50 km) to match the resolution of the climate data. Further environmental variables such as Enhanced vegetation index (EVI; MOD13C2[56]) and LAI (leaf area index) based land type (MCD12C1[57]) were also included. The indices for ecological facets were taken from maps published by the United States Geological Survey (USGS), which consider climate and community composition[58]. To better align with the definition of global mountains described in the main text, we then analyzed topographic and climatic data based on these "expert-identified" mountains[59] at global and regional scales (with different biogeographic realms[60] and cloud forests[63]. The spatial resolutions and temporal span of each dataset can be found in Extended Data Table 1.

Machine learning in analyzing biological data

We employed the 'RandomForestRegressor' ensemble method[64] from the 'sklearn' Python

package (v0.22.2.post1)[65] to model the relationship between the explanatory variables—Temperature rate and Moist ALRT—and the predictor variable, velocity, under conditions of low and high water vapor, respectively. Each ensemble consisted of 500 decision trees, each fitted with bootstrapped samples constituting 70% of the data. The random forest regressor algorithm assessed the importance of features (variables) based on their contribution to reducing impurity—specifically, the mean squared error between the predictions and actual values—at each split in the trees. The remaining 30% of the samples, not used in fitting the model (out-of-bag, OOB), were utilized to calculate the OOB score (an r-squared value), thereby evaluating model performance. Subsequently, we applied SHAP analysis using the 'TreeExplainer' function from the 'shap' package (v0.41.0) to further investigate the contribution of each variable to the prediction at the individual data point level.

Detailed taxonomic classification based on bioShift dataset

Taxonomic groups are classified mainly according to Chen *et al.*[3]. For plants, this taxonomic group includes species within the kingdom Plantae. For gastropods, species in the class Gastropoda are included; for insects, species in the class Insecta; for mammals, species in the class Mammalia; for birds, species in the class Aves; for reptiles, species in the class Reptilia; for amphibians, species in the class Amphibia.

Supplementary Results

The results showing MALR can better explain biological dataset

We used the random forest method of machine learning to calculate the relationship between MALRT and SLRT. After removing the top and bottom 2% of extreme values in the satellite data, we found that MALRT and SLRT are highly correlated. We can explain 44.55% of the variation in SLRT with just the longitude and latitude of the location and MALRT. Among them, MALRT can explain 29% of the explainable variation, showing a certain degree of consistency between the two. We also employed the random forest method to investigate the possible factors affecting species range shifts, including MALRT, SLRT, surface temperature, water vapour, precipitation, cloud cover, biological taxa, and methodological differences (e.g. sampling position [e.g. centroid, leading, or trailing edge], data type [occurrence or abundance], and spatial grain); see Supplementary Methods. The model decently predicted upward ($R^2 = 0.17$)

and downward ($R^2 = 0.18$) shifts in species distribution ($R^2 = 0.02$ for overall distribution shift; Extended Data Fig. 5c). Notably, upward and downward shifts had similar high-impact environmental variables. MALRT was the dominant factor with a higher average percentage of explanation (upward shift, 11.85%; downward shift, 19.29%; joint, 3.72%) in models with high predictability (upward and downward shifts), followed by cloud cover (upward shift, 21.31%; downward shift, 5.37%; joint, 20.34%; Extended Data Fig. 5c). Though sampling position was listed as the second dominant variable in the model with the lowest predictability ($R^2 = 0.02$) on the overall distribution shift, no methodological variable was identified more than once as dominant factors (explaining more than 5%). Both upward and downward shifts showed a strong association with abiotic conditions. The exact mechanism requires further investigation; nevertheless, the bidirectional nature of range shifts partially explains the low explanatory power in the joint database. SLRT explained a limited amount of variation (upward shift, 0.59%; downward shift, 0.37%; joint, 2.81%). We speculate that MALRT performs better because its calculations consider the effect of water vapour on air parcels in changing elevations and are based on CRU, including satellite observations, in situ climate records, and general circulation model simulations[42,43]. In contrast, the calculation of SLRTs relies only on satellite observations.

Supplementary Figures

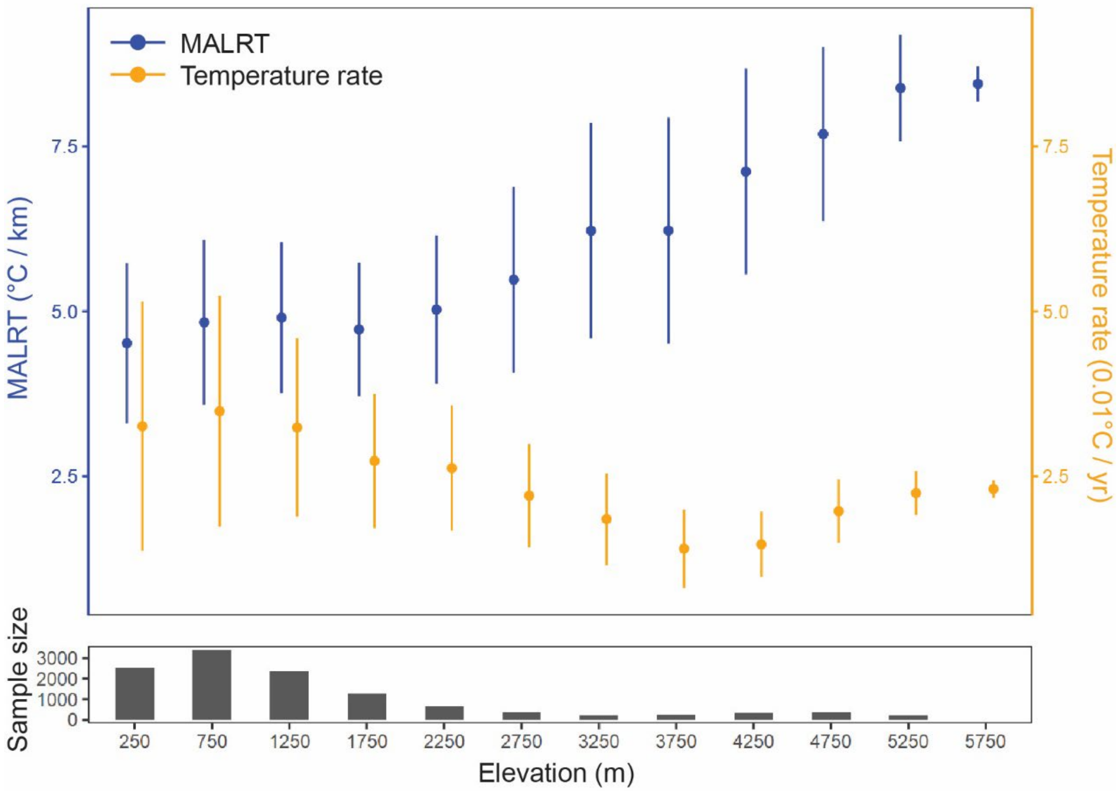

Supplementary Figure 1. MALRT and temperature rate along elevation. The centre and the error bars indicate mean and standard deviation, respectively. Sample size is provided in the lower panel.

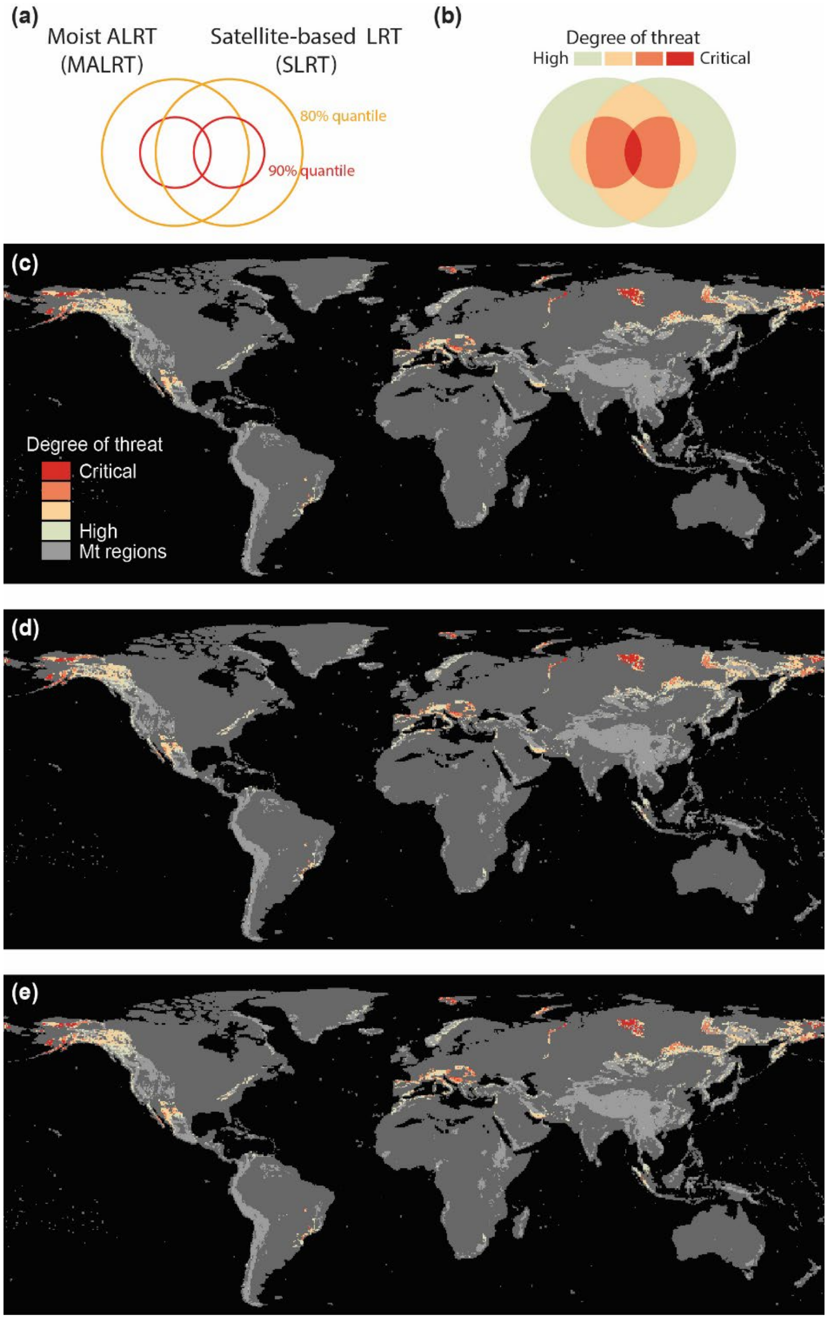

Supplementary Figure 2. Identifying mountain regions threatened by high vertical velocities with different outlier removal levels. A consensus map of the vertical velocities of isotherm shifts as estimated from the satellite-based lapse rate (SLRT) or from the moist adiabatic lapse rate (MALRT) (see Fig. 2). (a-c) Mountain regions where velocities are greater than 80% quantile (i.e. retaining 20%) in either calculation of MALRT or SLRT are labelled as critically threatened (a-b) and displayed in red (c-e). To address potential data artefacts, varying percentages of extremely low absolute SLRT values were excluded: (c) 0.5%, (d) 2%, and (e) 5%. For reference, the outcomes upon excluding 1% of these outlier SLRT values are detailed in Fig. 3.

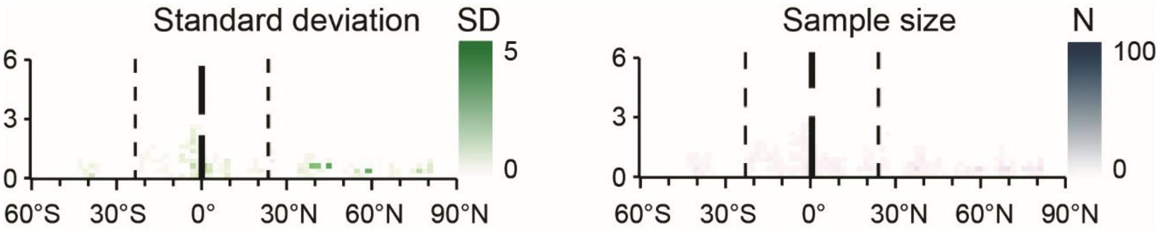

Supplementary Figure 3. The statistical details corresponding to Fig. 4d. The sample size and standard error are shown.

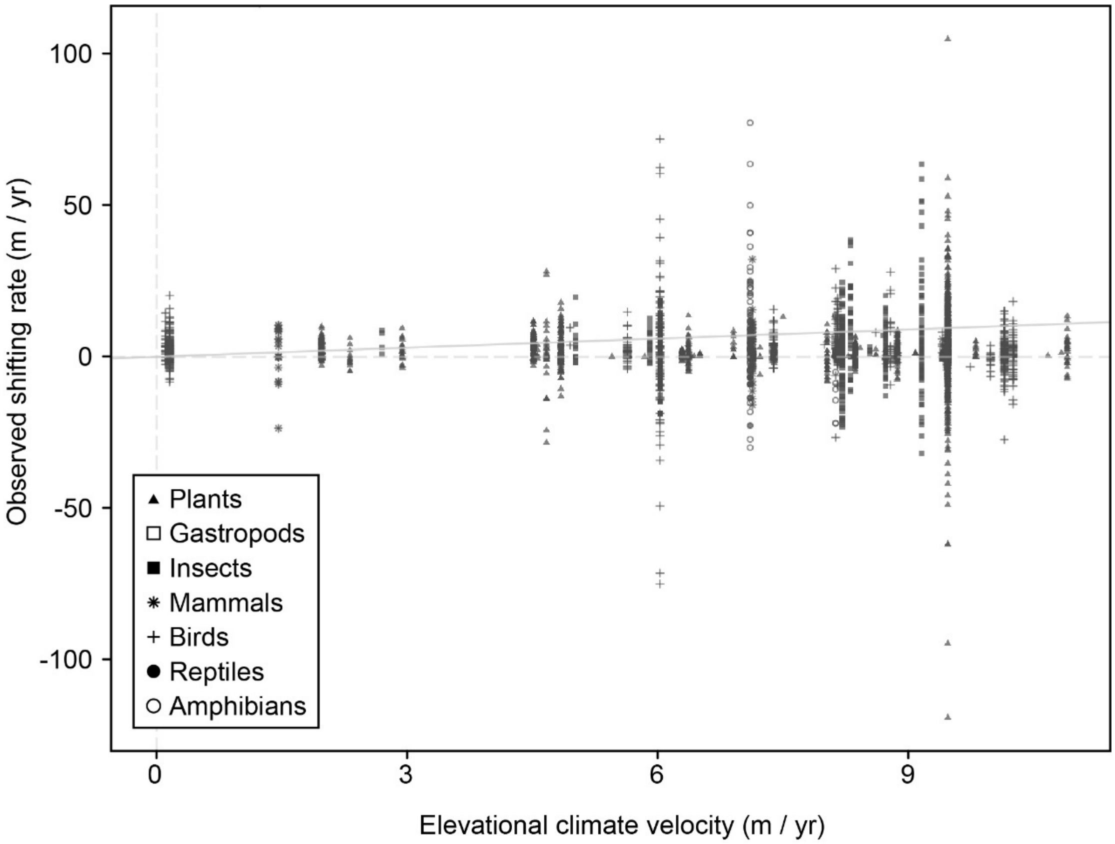

Supplementary Figure 4. Raw biological data points used in our analyses. All data points are shown. Since a proportion of data was collected from near localities at the global scale, many data points are highly overlapped.

61 Staver, A. C., Archibald, S. & Levin, S. A. The global extent and determinants of savanna and forest as alternative biome states. *Science* **334**, 230-232 (2011).
62 Wang, S. *et al.* Recent global decline of CO2 fertilization effects on vegetation photosynthesis. *Science* **370**, 1295-1300 (2020).
63 Wilson, A. M. & Jetz, W. Remotely Sensed High-Resolution Global Cloud Dynamics for Predicting Ecosystem and Biodiversity Distributions. *PLoS Biol* **14**, e1002415 (2016).
64 Breiman, L. Random forests. *Machine learning* **45**, 5-32 (2001).
65 Pedregosa, F. *et al.* Scikit-learn: Machine learning in Python. *the Journal of machine Learning research* **12**, 2825-2830 (2011).
