# Supplementary material for: Climate velocities and species tracking in global mountain regions
Source: Nature. 2024 Mar 27;629(8010):114–20. doi: 10.1038/s41586-024-07264-9 (PMC11062926; doi:10.1038/s41586-024-07264-9)
Supplement: Supplementary file 2 — Reporting Summary [file 41586_2024_7264_MOESM2_ESM.pdf]

nature portfolio

Corresponding author(s): Sheng-Feng Shen; I-Ching Chen

Last updated by author(s): Jan 15, 2024

Reporting Summary

Nature Portfolio wishes to improve the reproducibility of the work that we publish. This form provides structure for consistency and transparency in reporting. For further information on Nature Portfolio policies, see our Editorial Policies and the Editorial Policy Checklist.

Statistics

For all statistical analyses, confirm that the following items are present in the figure legend, table legend, main text, or Methods section.

n/a	Confirmed	
☒	☐	The exact sample size (*n*) for each experimental group/condition, given as a discrete number and unit of measurement
☒	☐	A statement on whether measurements were taken from distinct samples or whether the same sample was measured repeatedly
☐	☒	The statistical test(s) used AND whether they are one- or two-sided<br>*Only common tests should be described solely by name; describe more complex techniques in the Methods section.*
☒	☐	A description of all covariates tested
☒	☐	A description of any assumptions or corrections, such as tests of normality and adjustment for multiple comparisons
☐	☒	A full description of the statistical parameters including central tendency (e.g. means) or other basic estimates (e.g. regression coefficient) AND variation (e.g. standard deviation) or associated estimates of uncertainty (e.g. confidence intervals)
☐	☒	For null hypothesis testing, the test statistic (e.g. F, t, r) with confidence intervals, effect sizes, degrees of freedom and P value noted<br>*Give P values as exact values whenever suitable.*
☒	☐	For Bayesian analysis, information on the choice of priors and Markov chain Monte Carlo settings
☐	☒	For hierarchical and complex designs, identification of the appropriate level for tests and full reporting of outcomes
☒	☐	Estimates of effect sizes (e.g. Cohen's *d*, Pearson's *r*), indicating how they were calculated

Our web collection on statistics for biologists contains articles on many of the points above.

Software and code

Policy information about availability of computer code

Data collection	R4.0.4; Python 3.7.9
Data analysis	R4.0.4; Python 3.7.9; Wolfram Mathematica 12; sklearn v022.2.post1; shap v0.41.0

For manuscripts utilizing custom algorithms or software that are central to the research but not yet described in published literature, software must be made available to editors and reviewers. We strongly encourage code deposition in a community repository (e.g. GitHub). See the Nature Portfolio guidelines for submitting code & software for further information.

Data

Policy information about availability of data

All manuscripts must include a data availability statement. This statement should provide the following information, where applicable:

- Accession codes, unique identifiers, or web links for publicly available datasets
- A description of any restrictions on data availability
- For clinical datasets or third party data, please ensure that the statement adheres to our policy

The authors declare that the data supporting the findings of this study are available within the paper, its Extended Data Table and at https://doi.org/10.5061/dryad.1rn8pk0wm.
CRU TS4.05 at https://crudata.uea.ac.uk/cru/data/hrg/
MOD11C1 at https://lpdaac.usgs.gov/#nav-heading

MOD11C2 at https://lpdaac.usgs.gov/#nav-heading
MOD11C3 at https://lpdaac.usgs.gov/#nav-heading
EarthEnv at https://www.earthenv.org/
ETOPO1 at https://www.ncei.noaa.gov/products/etopo-global-relief-model
SRTM at https://www2.jpl.nasa.gov/srtm/
GMBA at https://www.gmba.unibe.ch/services/tools/mountain_inventory_v1
CHELSA at https://chelsa-climate.org/
GHCN at https://www.drought.gov/data-maps-tools/global-historical-climatology-network-ghcn
BioShifts at https://doi.org/10.6084/m9.figshare.7413365.v1

Research involving human participants, their data, or biological material

Policy information about studies with human participants or human data. See also policy information about sex, gender (identity/presentation), and sexual orientation and race, ethnicity and racism.

Reporting on sex and gender	N/A
Reporting on race, ethnicity, or other socially relevant groupings	N/A
Population characteristics	N/A
Recruitment	N/A
Ethics oversight	N/A

Note that full information on the approval of the study protocol must also be provided in the manuscript.

Field-specific reporting

Please select the one below that is the best fit for your research. If you are not sure, read the appropriate sections before making your selection.

☐ Life sciences ☐ Behavioural & social sciences ☒ Ecological, evolutionary & environmental sciences

For a reference copy of the document with all sections, see nature.com/documents/nr-reporting-summary-flat.pdf

Ecological, evolutionary & environmental sciences study design

All studies must disclose on these points even when the disclosure is negative.

Study description	The result data and figures presented in this paper are derived from published maps and datasets.
Research sample	A comprehensive list of data sources can be found in Extended Data Table 1. The mountain regions have been defined by experts, and the specific definition of islands can be found in the Methods section.
Sampling strategy	In general, all available data pixels were included in our analyses. Specific details for certain statistics, such as bootstraps, can be found in the Methods section, where they are further elaborated upon.
Data collection	Please find Extended Data Table 1 for details.
Timing and spatial scale	Our focus was on global mountain data spanning the periods of 1971-1980 and 2011-2020.
Data exclusions	Given that SLRT values approaching 0 can induce extremely high climate velocities, we excluded the 1% of outliers proximate to zero when generating Fig. 3c. In determining the relationship between MALRT and SLRT, we omitted 2% of the extreme values from the satellite data, encompassing both the upper and lower extremities. Furthermore, two conspicuous outliers derived from the weather station data were excluded before conducting the linear regression. Although incorporating these outliers might have enhanced the explanatory power of the weather station data, we surmise that such a result could be an artifact. As such, we opted to exclude these two outliers.
Reproducibility	The procedure has been repeated three times, and the results have consistently yielded similar outcomes, except for the steps involving randomization, which exhibit consistent but slightly different results.
Randomization	Randomization was incorporated into the procedure for calculating SLRT and in comparing biological range shift with MALRT. Further details regarding this randomization process can be found in the Methods section and Extended Data Figure 6.
Blinding	The concept of blinding treatment is not applicable in this context, as the analytical pipeline used for environmental datasets is unlikely to be influenced.

Did the study involve field work? ☐ Yes ☒ No

Reporting for specific materials, systems and methods

We require information from authors about some types of materials, experimental systems and methods used in many studies. Here, indicate whether each material, system or method listed is relevant to your study. If you are not sure if a list item applies to your research, read the appropriate section before selecting a response.

Materials & experimental systems

n/a	Involved in the study
☒	☐ Antibodies
☒	☐ Eukaryotic cell lines
☒	☐ Palaeontology and archaeology
☒	☐ Animals and other organisms
☒	☐ Clinical data
☒	☐ Dual use research of concern
☒	☐ Plants

Methods

n/a	Involved in the study
☒	☐ ChIP-seq
☒	☐ Flow cytometry
☒	☐ MRI-based neuroimaging
