# Supplementary figures and images for: Climate velocities and species tracking in global mountain regions
Source: Nature. 2024 Mar 27;629(8010):114–20. doi: 10.1038/s41586-024-07264-9 (PMC11062926; doi:10.1038/s41586-024-07264-9)

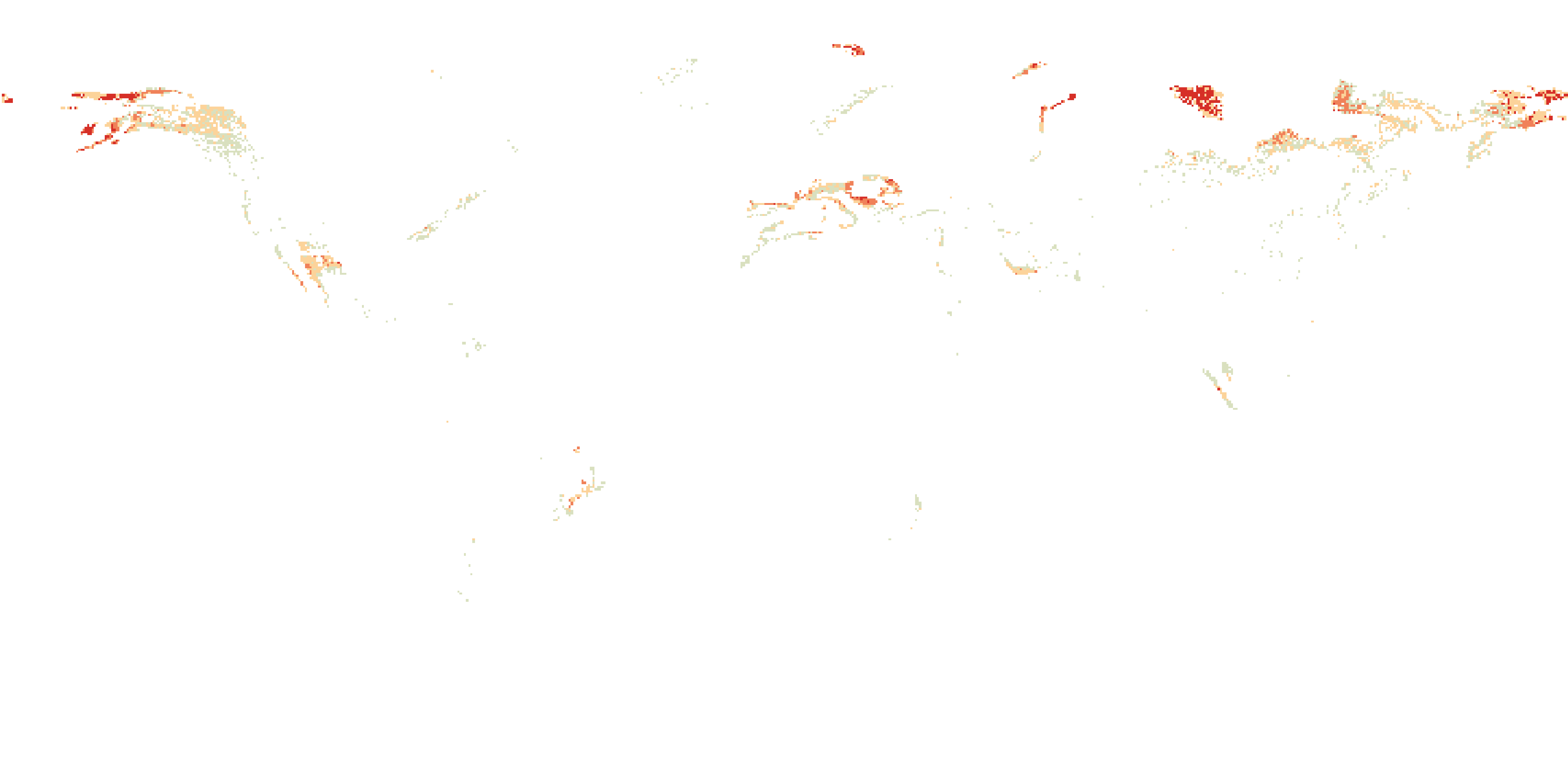

Supplement: Supplementary file 6 — A Google Earth layer file (*.kmz) that enables self-exploration. [file 41586_2024_7264_MOESM6_ESM.zip › Layer2.png]
